# Intravenous Treprostinil in Severe Inoperable Chronic Thromboembolic Pulmonary Hypertension Using Implantable Pumps—Single-Center Experience over More Than a Decade

**DOI:** 10.3390/jcdd10080318

**Published:** 2023-07-27

**Authors:** Regina Steringer-Mascherbauer, Charlotte Huber, Uwe Fröschl, Dagmar Engleder, Reinhold Függer, Maria Lummersdorfer, Ralf Lenhard, Martin Martinek

**Affiliations:** 1 Department of Cardiology, Ordensklinikum Linz GmbH Elisabethinen, 4020 Linz, Austria; charlotte.huber@ordensklinikum.at (C.H.);; 2 Department of Surgery, Ordensklinikum Linz GmbH Elisabethinen, 4020 Linz, Austria; uwe.froeschl@ordensklinikum.at (U.F.); reinhold.fuegger@ordensklinikum.at (R.F.); 3 Institute for Anesthesiology and Intensive Care, Ordensklinikum Linz GmbH Elisabethinen, 4020 Linz, Austria; 4 OrphaCare GmbH, 1190 Vienna, Austria; ralf.lenhard@orphacare.com

**Keywords:** chronic thromboembolic pulmonary hypertension, implantable pump, intravenous treprostinil

## Abstract

The management of chronic thromboembolic pulmonary hypertension has significantly changed over the last decade with the availability of both specific therapies and interventional treatments. In parallel, implantable pumps for intravenous administration of treprostinil have broadened the spectrum of continuous prostanoid infusion. We evaluated the course of 17 consecutive patients with inoperable chronic thromboembolic pulmonary hypertension treated with treprostinil by means of an implantable infusion pump between 2011 and 2023 at our center. Complications associated with the infusion system were rare, leading to 0.4 unplanned surgical interventions during 17,160 patient days. No additional safety signals were detected, and clinical benefits achieved with subcutaneous treprostinil before pump implantation could be maintained in all patients. No catheter-related infections or thromboembolic events were observed. Implantable infusion pumps offer an attractive alternative to subcutaneous treprostinil for patients intolerant to the subcutaneous route, including those with chronic thromboembolic pulmonary hypertension.

## 1. Introduction

Chronic thromboembolic pulmonary hypertension (CTEPH) is a type of pulmonary hypertension (PH) developing secondary to peripheral venous clot embolization and subsequent intravascular thrombosis [1], classified as Group 4 PH, pulmonary hypertension associated with chronic pulmonary artery obstruction. As chronic thromboembolic pulmonary hypertension is a common and important cause of PH and the therapeutic management strategy is significantly different from other forms of PH, current guidelines emphasize that the possibility of CTEPH should be carefully considered in all patients with PH, not only in patients with a history of pulmonary embolism but also taking into account other risk factors for CTEPH such as permanent intravascular devices, inflammatory bowel diseases, myeloproliverative neoplasms like essential thrombocythaemia or polycythaemia vera, splenectomy, antiphospholipid syndrome, high-dose thyroid hormone replacement, and malignancy. Despite the theoretical advantages of alternative perfusion imaging methods, ventilation/perfusion scanning is still the most effective technique to exclude chronic thromboembolic pulmonary disease (CTEPD). Importantly, a negative computed tomography pulmonary angiography with bi-planar reconstruction does not exclude CTEPD, as distal disease may remain undiagnosed [2]. As for pulmonary arterial hypertension, right heart catheterization (RHC) is required to confirm the diagnosis of CTEPH and assess the severity of haemodynamic impairment [3]. RHC should comprise a complete set of haemodynamics and is recommended to be performed following standardized protocols [2].

The therapeutic armamentarium for CTEPH has dramatically changed during the last 15 years, as clearly illustrated by the respective guidelines. While pulmonary endarterectomy (PEA) was already considered the treatment of choice for CTEPH and the only potential curative option in the 2009 guidelines, data on the benefits of PAH-specific drugs in CTEPH were incomplete and controversial, thus offering no strong recommendations for specific treatment in patients with severe inoperable CTEPH beyond individual assessment and treatment decisions at specialized centers [4]. In the following years, positive clinical trials led to European marketing authorizations for riociguat (Adempas^®^, Bayer AG, Basel, Switzerland) and treprostinil (Trepulmix^®^, SciPharm Sàrl, Mertert, Luxemburg) in 2014 and 2020, respectively [5,6]. In parallel, balloon pulmonary angioplasty emerged as an additional therapeutic option and is now considered an established treatment for selected patients with inoperable CTEPH or persistent/recurrent PH after PEA, improving haemodynamic right heart function, and exercise capacity with promising long-term outcomes, although long-term data are still scarce [2]. 

Treprostinil is a stable prostacyclin analogue with an elimination half-life of 4.5 h [7]. It has been approved in the majority of European countries for more than 15 years for idiopathic and hereditary pulmonary hypertension and has been shown to be effective with both subcutaneous (SC) and intravenous (IV) administration [8,9,10,11,12]. Continuous administration of treprostinil using external pumps, however, has drawbacks for both administration routes. While SC administration is associated with painful infusion site reactions in the vast majority of patients [8,9,10], catheter-related infections are a rare but possibly fatal complication of IV prostanoid administration [13]. Thus, the availability of a fully implantable pump for IV treprostinil in 2009 (Lenus pro^®^, Tricumed Medizintechnik GmbH, Kiel, Germany) provided a promising option to administer treprostinil while avoiding the local side effects of SC treatment and, in parallel, minimizing the risk for catheter-related infections. However, this approach requires surgical intervention, which is associated with significant risk for patients with PH [14]. At our center, an interdisciplinary expert panel consisting of physicians and nursing staff defined criteria and standards for the pre-, peri-, and postoperative management of patients undergoing this procedure. The first pump was implanted in 2010 at our center, and the first implantation in a patient with CTEPH was performed by mid-2011. Meanwhile, we have implanted more than 100 pumps in total at our center. Here we report our experience with implantable pumps for CTEPH.

## 2. Materials and Methods

Data on pulmonary hypertension patients were collected in the Elisabethinen Linz Pulmonary Hypertension Registry (ELPHREG) at our center. ELPHREG was approved by the concerned ethics committee, and written informed consent was obtained by all patients before inclusion in the registry. We performed a database analysis of all CTEPH patients who transitioned to IV treprostinil administered by an implantable infusion pump at our center between September 2010 and April 2023. 

Preoperative management of the patients followed our standards developed for patients diagnosed with PAH [15,16]: Treprostinil was initiated subcutaneously and uptitrated under close supervision of specialized nursing staff in an outpatient setting. Both uptitration and dose adjustments during long-term treatment were based on signs and symptoms of PH as reported both by the patient and responsible nursing staff as well as results obtained at control visits. As in PAH patients, implantation of an infusion pump was only considered for patients with significant side effects associated with the SC administration route, such as severe site pain or repeated site infections despite clinical response to treprostinil treatment, or as an alternative for patients unable to handle an external pump and having no reliable access to caretaking, as well as patients suffering from the stigma of wearing an external, visible infusion pump. Patients considered candidates for pump implantations were independently assessed by the treating PH physician, the anesthesiologist, and the surgeon, and in cases of significant comorbidities, the respective medical specialist was consulted. PH-specific therapy and concomitant medication were optimized prior to pump implantation. 

Implantation procedures were exclusively performed under general anesthesia, including extended hemodynamic monitoring. Both the anesthesiologic and surgical teams consisted of dedicated senior physicians. During the implantation of the pump, SC treprostinil treatment was continued. In a first step, a central venous access was placed either in the Vena subclavia or cephalica. After preparation of the pump pocket in the abdominal wall, the pump was filled by the surgeon with the calculated volume of treprostinil diluted with normal saline to completely fill the pump reservoir. As subcutaneously and intravenously administered treprostinil has been shown to be bioequivalent [7], the transition to IV administration was calculated on a 1:1 basis. A second catheter was fixed to the pump outlet. The pump was placed in the pump pocket, the catheter was tunneled towards the central venous access, and the two catheters were connected. Based on the experience in the first PAH patient implanted [16], we did not calculate the catheter volume as recommended as a basis for calculating the time the pump would need to fill the catheter with medication but stopped SC administration 60 min after the connection of the intravenous catheters in all patients to avoid an overlap and overdose effects. Postoperatively, blood pressure, oxygen saturation, and heart rate were continuously monitored for 24 h. All pump refills were done by dedicated, specifically trained staff at our outpatient clinic.

Descriptive data analysis was performed with Microsoft Excel 2019 (Microsoft Corp., Redmond, WA, USA). Continuous variables were summarized with the following descriptive statistics: N (number of observations), (arithmetic) mean, range: minimum (min)-maximum (max). Categorical variables were summarized using counts and percentages.

## 3. Results

**Figure 1:**
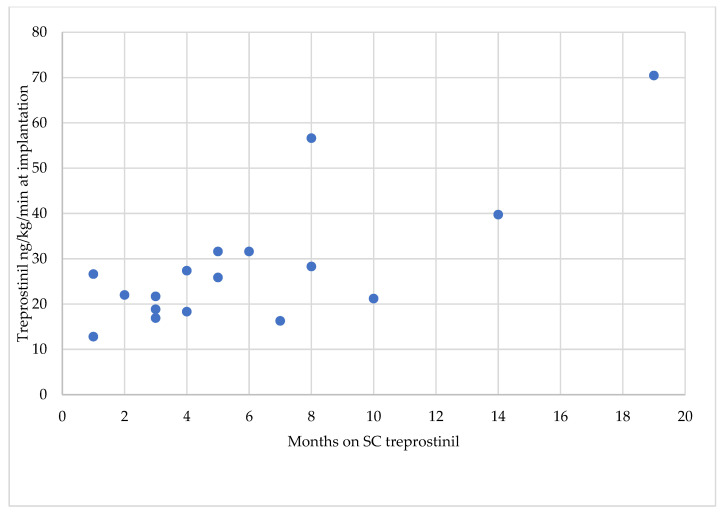
Months on SC treprostinil.

During the observation period, 17 consecutive patients (10 females and 7 males) diagnosed with severe inoperable CTEPH underwent pump implantation at our center. The mean age at the time of implantation was 71 years (range 43–82), the mean time on SC treprostinil before implantation was 6.1 months (range 1–19), and the mean treprostinil dose at implantation was 28.6 ng/kg/min (range: 12.8–70.45 ng/kg/min), as shown in Figure 1. Following our standard protocol, treprostinil was administered via SC infusion in all patients. The reason for switching to IV treprostinil was significant site pain in 16 (94.1%) patients and psychological reasons in 1 (5.9%) patient. Baseline characteristics are summarized in Table 1. All patients experienced significant clinical benefit with SC treprostinil, which could be maintained with IV administration.

In 4 (23.5%) patients, treprostinil was used as a first-line specific treatment; 10 (58.8%) received treprostinil on top of first-line oral monotherapy; and 3 (17.5%) patients added it to double oral therapy. The mean time from diagnosis to initiation of treprostinil was 28.6 months (range 1–84). At the time of initiation of treprostinil, patients were exclusively in WHO FC III (*n*= 14, 82.4%) and IV (*n* = 3, 17.6%) with a mean PAP of 47.24 mmHg (range 37–63) and a mean PVR of 8.8 WU (range 4–19.6). 

Two patients were initiated on SC treprostinil in the CETREPH trial [3] and switched to commercially available treprostinil (Remodulin^®^, Ferrer Internacional SA, Barcelona, Spain) after the end of the study. When the treprostinil formulation used in the CTREPH trial was licensed as a generic for PAH in Austria in 2018 (Trisuva^®^, Amomed Pharma GmbH, Vienna, Austria, now AOP Orphan Pharmaceuticals GmbH, Vienna, Austria), we gradually switched all our patients treated with treprostinil, including the CTEPH patients, to this formulation and also initiated all new patients on the generic drug.

Not surprisingly, for a real-life CTEPH population, most of our patients were diagnosed with significant comorbidities. Only two patients (11.8%) had no comorbidity, while nearly half of the patients (*n*= 8, 47.1%) were diagnosed with more than two significant medical conditions in addition to CTEPH, including malignancy in three (17.6%) patients. Two of these patients were diagnosed with hematological malignancies (T-cell lymphoma and chronic myelomonocytic leukemia), and the third with breast cancer.

At the time of evaluation, the mean follow-up time was 33.7 months (range 11–113), and the mean dose at the last follow-up was 57.1 ng/kg/min (range 12.6–205.1 ng/kg/min). Eleven (64.7%) patients died during the observation period; the mean time from implantation to death was 23.5 months (range 12–60). The causes of death were right heart failure and sudden cardiac death in four and two patients, respectively. In three patients, death was related to concomitant malignancy. One patient died in palliative care after a thigh fracture; a second patient died after a hip replacement.

No intraoperative complications were observed, and postoperatively, a case of hemothorax was successfully managed in the patient with concomitant T-cell lymphoma. In total, twelve surgical interventions related to the pump system were performed in nine patients during 17,160 patient days, including five planned surgeries and seven unplanned interventions for system-related reasons. In two cases, corrective surgery was performed to correct catheter dislocations with mechanical damage to the catheter leading to subcutaneous leakage and the respective local site reaction. In both cases, abrupt overhead movements were identified as likely causes. Abnormally high reflow at pump refill, indicating reduced drug delivery, led to surgical intervention in four patients. Of note, none of these patients demonstrated clinical signs of underdosing or worsening of CTEPH. Mechanical damage to the catheter could be identified by imaging and confirmed intraoperatively, subsequently leading to the change of the catheter only in two patients, whereas in the remaining two patients no definite reason could be identified during surgery. Therefore, both the pump and catheter were changed in these patients. External examination of the implanting pumps revealed normal pump function, indicating some non-visible catheter damage as the reason for increased reflow. One patient developed a surgical site infection after the change of the catheter, leading to the decision to explant the infusion pump and re-initiate SC treprostinil. Four months later, a new pump was implanted in this patient as a planned implantation.

A gradual increase in flow rate in the long-term use of implanted pumps with treprostinil has already been observed in the first years after the availability of the implantable pumps, leading to shorter refill intervals and, ultimately, the change of the infusion pump [15]. During the observation period, four pumps were replaced for increased flow rates at 38, 46, 52, and 63 months after implantation. 

All surgical interventions were performed without complications by the dedicated surgical team. During more than 600 refill procedures at our outpatient clinic, we did not observe a single complication. No catheter-related infection occurred during 17,160 patient days, nor do we have any evidence of thromboembolic events or clinical deterioration related to the pump system.

## 4. Discussion

The therapeutic approach to CTEPH has fundamentally changed since 2010, when only PEA was available as an established treatment option for patients considered technically operable and specific treatments were offered at expert centers on a case-by-case basis. When we took the decision to offer continuous parenteral prostanoid treatment to patients with inoperable CTEPH in 2011, no specific therapy was approved for this indication. While the BENEFiT trial, at that time the only randomized controlled trial, delivered inconclusive results [17], several uncontrolled series indicated a positive effect of phosphodiesterase V inhibitors [18] and prostanoids [19,20]. In particular, Skoro-Sajer and coworkers [20] delivered promising results in both clinical and hemodynamic endpoints using SC treprostinil. While we clearly could confirm the clinical benefits of SC treprostinil in patients with inoperable CTEPH, not surprisingly, the patients also experienced site pain associated with the change of the infusion site. Although this can be observed in almost every single patient, the majority of patients are able and willing to accept this effect, especially if, as in our case, close support for patients is also provided by specialized nursing staff outside the hospital setting. For patients significantly suffering from site pain, however, the availability of implantable pumps for IV treprostinil therapy in 2009 and, at our center, 2010 offered an important additional therapeutic option. Central venous access systems are associated with thrombotic complications, specifically in patients with a history of thromboembolic disease [21]. Furthermore, there is insufficient evidence that central venous access systems are a risk factor for the development of CTEPH [22]. However, a French group demonstrated not only the efficacy but also the feasibility and safety of long-term IV epoprostenol in CTEPH [23]. As treprostinil, for its pharmacological properties, is the prostanoid of choice at our center, and the course of the first PAH patients on the implantable pump was encouraging, we decided to also propose this option to patients with CTEPH and severe site pain with SC treprostinil. As for PAH as well, we consider it of utmost importance to carefully weigh the benefits and risks of both continuing SC therapy and performing surgery for pump implantation. For us, it is equally important to thoroughly prepare the patient in an interdisciplinary way and optimize specific and concomitant medications prior to the procedure. We are convinced that this strict approach is heavily contributing to the low observed complication rate at our center. 

The 13 observed procedure- or device-related incidents, as shown in Table 2, correspond to 0.7 cases per 1000 patient days. Unplanned surgical interventions contributed 0.4; planned surgery, including pump changes for increased flow rate, contributed 0.29; and procedure-related events contributed 0.06 incidents per 1000 patient days. While this is numerically higher than in our whole cohort published in 2020 [15], a comparison of total incident rates does not make sense, as we have increasingly performed planned changes to implanted pumps for increased flow rate since 2021, not only in CTEPH patients. Currently, there are six patients listed for pump changes, two of whom have been diagnosed with CTEPH. Events leading to unplanned surgical interventions were mainly catheter-related; the observed incidence of 0.4 cases per 1000 patient days can be considered very low in a cohort of elderly multimorbid patients. 

The efficacy of parenteral prostanoids in general and treprostinil in CTEPH has been demonstrated in both retrospective series [19,20,23] and a large randomized controlled trial [6], which led to the European marketing authorization of treprostinil for CTEPH in 2020. Treprostinil was exclusively administered subcutaneously in all of these series. To the best of our knowledge, this is the first evaluation of CTEPH patients treated with intravenous treprostinil administered by means of an implantable pump. The clinical benefits observed in our cohort mirror the positive results previously published with subcutaneous treprostinil in CTEPH. Given the bioequivalence of the two administration routes, however, this is also not surprising.

The use of implantable pumps for IV treprostinil has been established as an important option in the routine management of patients with PH since 2009. The safety of both the implantation procedure and long-term treatment has been reported in large cohorts of patients [15,24]. We did not detect any new safety signals in our cohort of CTEPH patients. As for PAH, we strongly advocate the same structured, individualized approach to the preoperative management of patients, optimizing not only PH specific medication. As shown in Figure 1, this can mean several months on SC treprostinil for dose titration. We do not support the approach of implanting before a clinical response to treprostinil. 

Interpretation of our results is difficult because there are quite a few limitations to our study. Besides its retrospective nature, a selection bias cannot be excluded in a single-center setting. More importantly, however, the evolving treatment landscape for CTEPH has heavily influenced our therapeutic approach to our patients since 2011, when the first patient in our cohort was initiated on treprostinil. Besides the availability of licensed drugs, the availability of BPA significantly changed the therapeutic approach to the treatment of CTEPH, which is reflected by an IB recommendation in the current guidelines [2]. While the recommendation for a multimodal approach with IIb is weaker, we are strongly convinced that this will be the future of CTEPH management. To achieve this, however, the place of medical therapy in the context of BPA still needs to be defined. 

In conclusion, the efficacy and safety of SC treprostinil have been demonstrated in CTEPH. Treprostinil is the only medicinal product licensed for the treatment of CTEPH patients in functional class IV via the SC route. Intravenous administration of treprostinil by means of an implantable pump was safe, effective, and feasible in our cohort of patients with severe inoperable CTEPH. Device-related incidences requiring unplanned surgery were rare, with seven cases during 17,160 patient days. No case of a catheter-related infection or thromboembolic event was observed. The increase in flow rate of the implantable pumps over time needs to be observed carefully, and future development should aim to develop devices with improved flow-rate control.

## Figures and Tables

**Table 1:** Patient population baseline and demographic characteristics.

		Treprostinil Treatment Initiation Timepoint			IIP Implantation Timepoint		
Patient N°	Sex	mPAP [mmHg]	PVR [WU]	WHO FC	Age [Years]	Duration of SC Treprostinil Therapy [Months]	Treprostinil Dose [ng/kg/min]
1	f	47	4	III	56	5	31.6
2	m	37	13.5	IV	74	14	39.74
3	f	63	17	III	43	4	18.3
4th	f	46	4	III	80	8	28.28
5	m	44	5	IV	67	5	25.85
6	f	44	4	III	78	3	18.85
7	m	34	6	III	82	10	21.21
8	f	36	6	III	75	1	26.61
9	f	45	11	III	72	4	27.36
10	m	45	12	III	75	8	56.6
11	f	59	11	III	53	1	12.8
12	m	61	19.6	III	82	19	70.45
13	m	57	11	III	82	3	21.69
14	f	50	7.5	III	73	2	22
15	f	45	8	III	73	3	16.89
16	m	42	6	III	60	7	16.28
17	f	48	4	III	82	6	31.6

PVR: Pulmonary vascular resistance; mPAP: mean pulmonary artery pressure; WU: Woods units; WHO FC: World Health Organization functional classification; SC: subcutaneous; f: female; m: male.

**Table 2:** Summary of all complications.

Complications	Count	Per 1000 Patient Days
**Total**	**13**	**0.8**
**Procedure related**	**1**	**0.1**
Postoperative hospital stay	1	0.1
**Device related**	**12**	**0.7**
Planned		
Pump flow rate (planned change)	4	0.2
Related implantation	1	0.1
Unplanned		
Connector dislocation	2	0.1
Pump/catheter problem without pump change	2	0.1
Pump/catheter problem with pump change	2	0.1
Explantation	1	0.1

## Data Availability

At our center, data on pulmonary hypertension patients were collected in the Elisabethinen Linz Pulmonary Hypertension Registry (ELPHREG). The data presented in this study are available on request from the corresponding author.
